# CT imaging of ovarian yolk sac tumor with emphasis on differential diagnosis

**DOI:** 10.1038/srep11000

**Published:** 2015-06-15

**Authors:** Yang-Kang Li, Yu Zheng, Jian-Bang Lin, Gui-Xiao Xu, Ai-Qun Cai, Xiu-Guo Zhou, Guo-Jun Zhang

**Affiliations:** 1 Department of Radiology, Cancer Hospital, Shantou University Medical College, Shantou 515041, China; 2 Department of Clinical Pharmacology, Cancer Hospital, Shantou University Medical College, Shantou 515041, China; 3 State Key Laboratory of Oncology in South China, Department of Diagnostic Imaging and Intervening Center, Cancer Center of Sun Yat-sen University, Guangzhou 510060, China; 4 The Breast Center, Cancer Hospital of Shantou University Medical College, Shantou 515041, China; 5 Cancer Research Center, Shantou University Medical College, Shantou 515041, China

## Abstract

Ovarian yolk sac tumors (YSTs) are rare neoplasms. No radiological study has been done to compare the imaging findings between this type of tumor and other ovarian tumors. Here we analyzed the CT findings of 11 pathologically proven ovarian YSTs and compared their imaging findings with 18 other types of ovarian tumors in the same age range. Patient age, tumor size, tumor shape, ascites and metastasis of two groups did not differ significantly (*P* > 0.05). A mixed solid-cystic nature, intratumoral hemorrhage, marked enhancement and dilated intratumoral vessel of two groups differed significantly (*P* < 0.05). The area under the ROC curve of four significant CT features was 0.679, 0.707, 0.705, and 1.000, respectively. Multivariate logistic regression analysis identified two independent signs of YST: intratumoral hemorrhage and marked enhancement. Our results show that certain suggestive CT signs that may be valuable for improving the accuracy of imaging diagnosis of YST and may be helpful in distinguishing YST from other ovarian tumors.

Yolk sac tumor (YST) of the ovary, also known as endodermal sinus tumor, is a primitive malignant germ cell tumor (GCT) that occurs in girls and young women. Descriptions of this tumor in radiological literature range from entirely solid to predominantly cystic^1^,^2^,^3^. However, these studies were case reports or usually lacked a comparative study with other ovarian tumors. Because the above radiological findings can also be found in many other types of ovarian tumors, a detailed radiological study of ovarian YST with emphasis on differential diagnosis is needed.

Characterization of an ovarian mass is of the utmost importance in the preoperative evaluation of an ovarian neoplasm. It can aid in surgical planning, whether exploration or laparoscopic excision, and may help distinguish benign from malignant tumors and thus avoid inappropriate management, or enable the surgeon to anticipate ovarian carcinoma preoperatively so that adequate procedures can be planned^4^.

By now, the combination of operation with chemotherapy remains the mainstay of therapy for YST, so familiarity of its radiological characteristics may permit preoperative diagnosis and improve surgical management of patients. Although similar radiological features can exist in YST and other types of ovarian tumors, predominant or specific imaging features may present in each type of tumor. Knowledge of these key imaging features may allow a specific diagnosis or substantial narrowing of the differential diagnosis, and may help distinguish YST from benign or other types of malignant ovarian tumors. In the present study, we not only demonstrate the radiological characteristics of ovarian YST on computed tomography (CT) images, but also focus on the differential diagnosis of this tumor.

## Methods

### Patients

Between August 2006 and October 2014, ten patients with pathologically proven primary ovarian YSTs of two institutions (Cancer Hospital of Shantou University Medical College and Cancer Center of Sun Yat-sen University) were identified. The database of our hospital (Cancer Hospital of Shantou University Medical College) was reviewed in order to identify control patients presenting in the same period with pathologically proven primary ovarian tumors. 16 patients with non-YST ovarian tumors were seleted. Inclusion criteria: (1) the age of the patient was below 30 years old; (2) the patient did not receive any treatment; and (3) each patient had complete CT data (plain scan and contrast-enhanced scan of abdominopelvic area).

Thus, twenty-six patients were included in the study. They were divided into 2 groups, including 10 patients in the YST group and 16 patients in the non-YST group. Bilateral ovarian YSTs were detected in 1 patient, and a total of 11 tumors were evaluated in the YST group. Bilateral ovarian cystadenocarcinomas were detected in 2 patients, and a total of 18 tumors were evaluated in the non-YST group, including 3 dysgerminoma, 2 immature teratomas, 9 serous or mucinous cystadenocarcinomas, 1 borderline serous cystadenoma, 1 struma ovarii, 1 Sertoli-Leydig cell tumor (SLCT) and 1 sclerosing stromal tumor (SST). Radiological characteristics, laboratory tests and operation notes of all cases were analyzed.

### CT examination and image analysis

CT examinations of 1 patient was performed on a PQ5000 spiral CT scanner (Picker, New York, NY, USA), 5 patients were examined using a Philips Brilliance TM 16-detector-row scanner (Philips Medical Systems, Cleveland, OH, USA), 20 patients were examined using a GE Brightspeed Elite 16-detector-row scanner (GE Healthcare, Milwaukee, WI, USA). After a series of unenhanced sections, all patients received intravenous bolus injection of contrast medium (Ultravist 300; Bayer Schering Pharma, Berlin-Wedding, Germany) at a rate of 2.5–3 mL/sec and a volume of 75–90 mL. The section thickness of all images of the single spiral CT was 10 mm. For multidetector CT, contiguous axial images and multiplanar reconstructions (MPR) were performed routinely. The section thickness was 5 mm and reconstruction interval was 1.25 mm. The examinations were replayed in abdomen (W = 250, L = 25).

Two radiologists who specialized in imaging of gynecological oncology retrospectively reviewed all images without knowledge of the patients’ clinical histories, laboratory tests, the results of the surgical resection and pathological results. Image reviews were done jointly and by consensus. The following CT features of each mass were assessed and recorded: tumor size (maximal diameter), shape (oval or irregular), margin (distinct or indistinct), density (entirely solid, predominantly solid, predominantly cystic or solid cystic), internal component (presence of hemorrhage, necrosis, calcification or fatty tissue), pattern of enhancement (homogeneous or heterogeneous), and degree of enhancement. Findings on CT images for ascites and metastasis (lymphadenopathy or peritoneal spread) were also evaluated.

Especially, the size was measured with the longest diameter of the biggest MPR section as criteria. The margin was split into distinct and indistinct according to the integrity of the tumor wall, or in terms of presence or absence of fat space between the mass and peripheral tissues and organs. The density was subjectively assessed and classified as predominantly solid, solid cystic and predominantly cystic, corresponding to the volume of cystic lesion less than 25%, between 25% and 75%, and greater than 75% of the tumor volume, respectively. The maximal length, width, and height of the lesion/tumor on MPR images were measured for volume calculation. When no obvious cystic lesions or solid lesions were seen in the tumor, the density was defined as entirely solid or entirely cystic, respectively. Intratumoral hemorrhage was defined as an amorphous hyperdense lesion with a relatively lower attenuation compared with calcification on noncontrast CT scans and no enhancement on contrast-enhanced CT scans. The degree of enhancement was subjectively assessed and categorized as follows: mild, when the enhancement was similar to that of adjacent muscle; moderate, when the enhancement was higher than that of muscle, but lower than that of blood vessels; and marked, when the enhancement was approaching that of blood vessels. Lymphadenopathy was defined as lymph nodes greater than 10 mm in short axis dimension.

Enlarged intratumoral vessel, also called “bright dot” sign in the literature about YST^3^, was specifically evaluated in the study and was recorded for each tumor. This sign was defined as different amount of dilated vessels in the tumor on post-contrast images. Besides conventional images, post-contrast axial MPR images were used to evaluate this sign. Axial MPR images could provide valuable information in some circumstances. For example, the density of the tumor parenchyma with marked enhancement was similar to that of vessels on post-contrast images, and “bright dot” sign might be overlooked in these areas. MPR images could detect the sign due to the thinner section thickness. Furthermore, in some cases, adjacent vessels were extremely displaced by the tumor and might mimic intratumoral vessels in peripheral tumor parenchyma. These vessels were usually tortuous and distorted, which could lead to difficulty in recognizing their origin or in displaying their path on conventional images. MPR images could track these vessels more clearly due to the thinner section thickness.

### Statistical analysis

Difference of the patient age and CT features between two groups was performed using the SPSS software package (version 17.0). Quantitative data were compared with *t*-test and qualitative data were compared with Chi-square test. *P* values of <0.05 were considered to be statistically significant. When the radiological signs appeared to be significant in the univariate analysis, multivariate analysis was performed for YST group using logistic regression model. The diagnostic performance of each sign was established using the area under the receiver operating characteristic (ROC) curve.

## Results

### YST group

**Figure 1:**
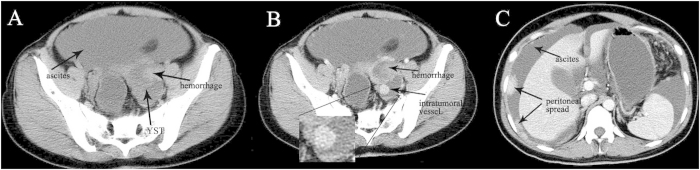
27-year-old young woman with ovarian yolk sac tumor. **A** Precontrast CT scan shows a high density lesion within a solid-cystic mass (black arrow). Massive ascites is seen (black arrow). **B** This high density lesion has no enhancement on post-contrast CT image, which indicates the lesion is hemorrhage (black arrow). Heterogeneous marked enhancement of the tumor and enlarged intratumoral vessels are detected (black arrow). **C** Multiple peritoneal metastases (black arrows) with marked enhancement and massive ascites (black arrow) are seen.

**Figure 2:**
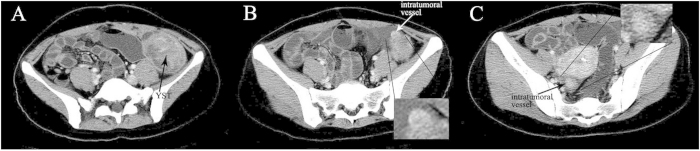
24-year-old young woman with bilateral ovarian yolk sac tumors. **A** A solid cystic mass with heterogeneous moderate to marked enhancement in the left pelvic cavity (black arrow). **B** An enlarged vessel is seen in the mass (white arrow). **C** The other solid cystic mass with an enlarged intratumoral vessel is seen in the right pelvic cavity (black arrow). Ascites is also detected.

**Figure 3:**
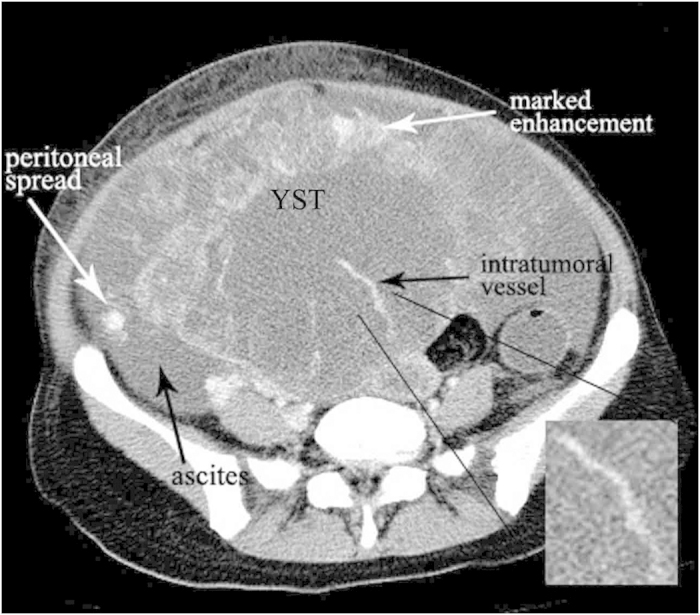
16-year-old girl with ovarian yolk sac tumor. A predominantly cystic mass with heterogeneous marked enhancement in the pelvic cavity (white arrow). Multiple enlarged vessels are seen in the mass (black arrow). Peritoneal metastases (white arrow) with marked enhancement and massive ascites (black arrow) are also seen.

The median age of diagnosis was 19.1 years for the total cases with a range of 15–27 years. Bilateral ovarian YSTs were detected in one patient. So a total of 11 tumors were analyzed. The size of the tumors ranged from 3.5 to 24.0 cm (mean, 13.9 cm). The shape was seen as oval (n = 8) or irregular (n = 3). All tumors showed a distinct margin. A mixed solid and cystic nature was seen in 7 tumors (Figs. 1 and 2). 1 tumor showed a completely solid mass, and 3 tumors showed a predominantly cystic mass (Fig. 3). Hemorrhage was seen in 7 tumors (Fig. 1A,B). Calcification was seen in 1 tumor. Ascites was seen in 8 patients (Figs. 1,2 and 3). Peritoneal spread or lymphadenopathy was seen in 6 patients (Figs. 1C and 3). On unenhanced CT images, the density of the solid component of all tumors was 34–52 HU (mean, 42 HU) which was similar to that of muscle. After contrast medium administration, there were heterogeneous marked enhancement in 7 (Figs. 1B,C and 3), heterogeneous moderate to marked enhancement in 3 (Fig. 2A,B) and heterogeneous mild to moderate enhancement in 1 at the solid portion of the lesions. Moreover, enlarged intratumoral vessels were seen in all tumors (Figs. 1B,2B,C and 3).

The level of preoperative serum alpha-fetoprotein (AFP) of all cases was over 1000 ng/ml (normal value ≤ 7 ng/ml). Intraoperatively, the tumors were completely removed and were encapsulated with a smooth, glistening external surface. Characteristically, the masses were cystic and solid with soft, gray-to-yellow tissue as well as areas of hemorrhage and necrosis on the cut sections. Under light microscope, all tumors had varying degrees of hemorrhage, necrosis or cystic degeneration with multiple enlarged vessels. Schiller-Duval body, a distinguishing feature of YST, was detected in all cases. Ascites, peritoneal spread or lymphatic metastasis on CT images were confirmed by surgery and pathology.

### non-YST group

**Figure 4:**
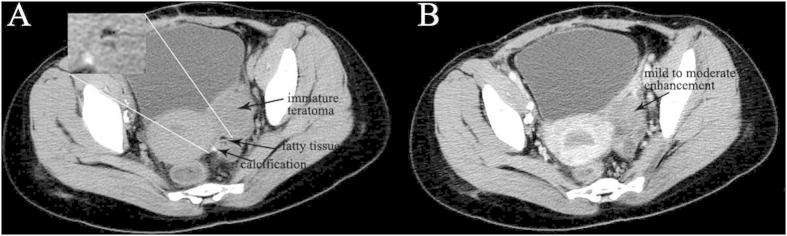
14-year-old girl with immature teratoma. **A** A solid-cystic mass with irregular shape is seen in the pelvic cavity. The tumor contains fatty tissues (black arrow) and calcification (black arrow) on precontrast image. **B** The tumor shows heterogeneous mild to moderate enhancement (black arrow).

**Figure 5:**
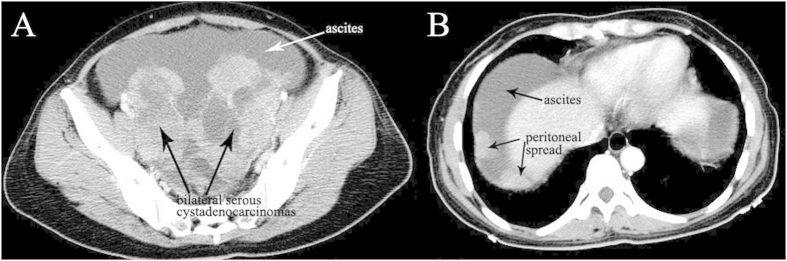
29-year-old woman with bilateral serous cystadenocarcinomas. **A** Bilateral ovarian masses (black arrows) with irregular shape, solid cystic nature, heterogeneous moderate to marked enhancement and ascites (white arrow) are seen in the pelvic cavity. **B** Multiple peritoneal metastases (black arrows) and massive ascites (black arrow) are seen.

**Figure 6:**
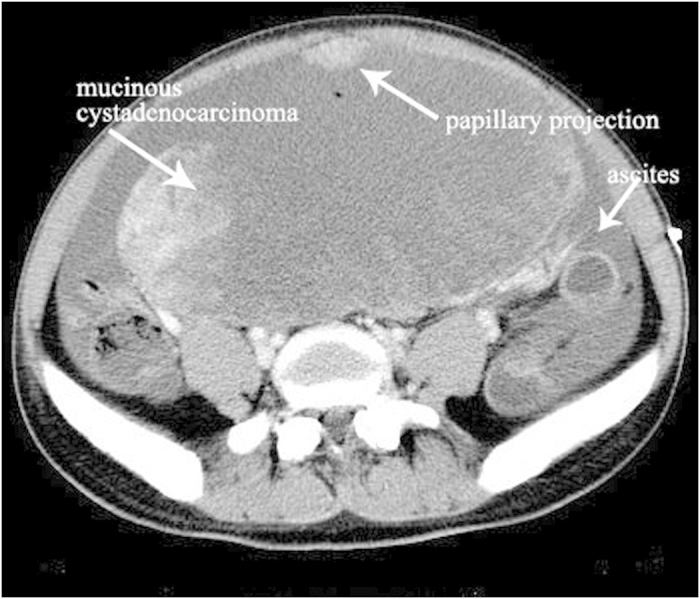
27-year-old young woman with mucinous cystadenocarcinoma. The tumor shows oval shape, predominantly cystic nature, heterogeneous moderate to marked enhancement (white arrow) and intratumoral papillary projection (white arrow). Ascites is also detected (white arrow).

**Figure 7:**
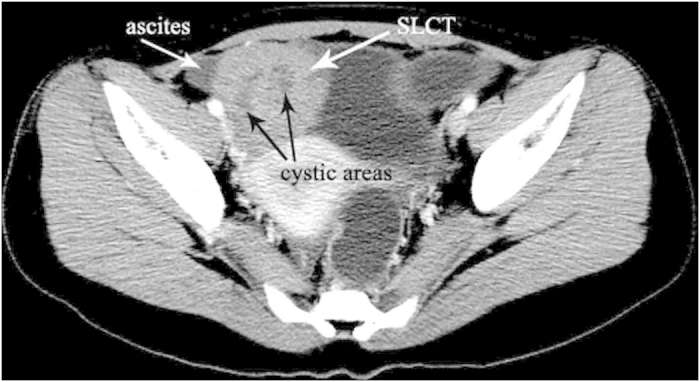
18-year-old woman with Sertoli-Leydig cell tumor. A predominantly solid mass contains little cystic areas (black arrows) in the right pelvic cavity. The tumor shows heterogeneous mild to moderate enhancement on post-contrast scan. Little ascites is also detected (white arrow).

The median age of diagnosis was 21.2 years for the total cases with a range of 14–29 years. Bilateral serous cystadenocarcinomas were detected in 2 patients. So a total of 18 tumors were analyzed. The size of all tumors ranged from 5.0 to 38.0 cm (mean, 14.6 cm). The shape was seen as oval (n = 7) or irregular (n = 11). All tumors showed a distinct margin. A mixed solid and cystic nature was seen in 5 tumors (Figs. 4 and 5A). 6 tumors showed a predominantly cystic mass (Fig. 6) and 7 tumors showed a predominantly solid mass (Fig. 6). Hemorrhage was seen in 4 tumors. Calcification was seen in 4 tumors (Fig. 4A). Fatty tissue was seen in 2 tumors (Fig. 4A). Ascites was seen in 13 patients (Figs. 5,6 and 7). Peritoneal spread or lymphadenopathy was seen in 7 patients (Fig. 5B). On unenhanced CT images, the density of the solid component of all tumors was 29–83 HU (mean, 49 HU). After contrast medium administration, there was no enhancement in 1, heterogeneous mild enhancement in 2, heterogeneous mild to moderate enhancement in 6 (Figs. 4B and 7), heterogeneous moderate to marked enhancement in 7 (Figs. 5 and 6) and heterogeneous marked enhancement in 1 at the solid portion of the lesions. 1 tumor showed heterogeneously marked ring enhancement and progressively centripetal enhancing. The sign of intratumoral vessel was absent in all tumors.

Increased serum level of CA125 was seen in all patients with epithelial carcinomas (range:159–4553 U/ml, mean:1521 U/ml). Increased serum level of androgen was seen in the patient with SLCT. All laboratory tests including tumor markers and serum hormonal assays were normal in all other tumors. Intraoperatively, all tumors were completely removed. Areas of intratumoral hemorrhage and necrosis were seen in all epithelial carcinomas on gross and microscopic studies. Ascites, peritoneal spread or lymphatic metastasis on CT images were confirmed by surgery and pathology.

### Statistical results

The statistical results of univariate analysis are summarized in Table 1. Patient age, tumor’ maximal diameter, tumor shape and ascites of two groups did not differ significantly (*P* > 0.05). When calculating the difference of metastasis (peritoneal spread or lymphadenopathy) between two groups, two cases with benign tumors (sclerosing stromal tumor and struma ovarii) were excluded from the non-YST group. The signs of metastasis between two groups also did not differ significantly (*P* > 0.05). Four CT features, including a mixed solid-cystic nature, intratumoral hemorrhage, marked enhancement and dilated intratumoral vessel of two groups differed significantly (*P* < 0.05). The area under the ROC curve of four significant CT features was 0.679, 0.707, 0.705, and 1.000, respectively.

An area of 1 represented that dilated intratumoral vessel was a perfect CT feature to diagnose YST. So this sign did not need to participate in multivariate analysis. According to multivariate analysis, two separate signs independently associated with YST were identified: the presence of intratumoral hemorrhage (*P* = 0.032) and marked enhancement (*P* = 0.045), whereas a mixed solid-cystic nature was not significant (Table 2).

## Discussion

YST is a rare tumor and comprises only 1% of all ovarian cancers. It is the third most common germ cell malignancy of the ovary accounting for about 20% of malignant germ cell tumors^5^. To our knowledge, this article is the largest study of ovarian YST in the radiological literatures.

In the present study, the CT findings of YST ranged from entirely solid to predominantly cystic or presented as heterogeneous appearances consisting of a mixed and cystic solid nature. The cystic areas are considered to be composed of epithelial line cysts produced by the tumor or of coexisting mature teratomas^6^. Most of the tumors in our study were large, oval with well-circumscribed margin, which may be associated with the origin of this kind of tumor. YSTs originate in germ cells not in the epithelium. They tend to grow in an expansive centripetal fashion and cause mass effect on surrounding structures. Adjacent organs are usually displaced, rather than infiltrated.

On unenhanced CT, the solid portion of YSTs appeared as isodense with normal muscle as the standard for comparison. A pattern of heterogeneous marked enhancement was present in most tumors in this study. Heterogeneity corresponds to intralesional hemorrhage, necrosis or cystic change. Marked enhancement reveals rich blood supply of the tumor parenchyma. Both were confirmed on cut sections and microscopic examinations. Intralesional hemorrhage may be caused by the hypervascularity of the tumor. Moreover, large tumor size may outgrow tumor blood supply and also cause intratumoral hemorrhage, necrosis or cystic degeneration. Awareness of intratumoral hemorrhage is recommended because it is difficult to identify in some cases. In our opinion, non-contrast CT examination is useful to demonstrate hyperdensity material that probably indicates active intratumoral hemorrhagic content. But CT has limitation to detect subacute and chronic hemorrhage or small areas of hemorrhage. Furthermore, differentiation necrosis or cystic changes within tumors from liquefied hemorrhage is also difficult. MR can provide complementary information regarding intratumoral hemorrhage, especially in the subacute and chronic stages^7^. Yamaoka *et al*. reported that three of four (75%) YSTs had intratumoral hemorrhage which showed high intensity on T1-weighted MR images^1^.

In the present study, the most characteristic CT finding of YSTs was enlarged intratumoral vessels on post-contrast images, which indicated the hypervascularity of YST. This finding was consistent to previous studies. Choi *et al*. reported that massive intratumoral vessels were observed in all 10 cases on post-contrast CT images and especially used the term “bright dot sign” to depict this CT finding^3^. Yomaoka *et al*. also reported that tumors showed a signal void on MR images and noted the presence of abundant vessels within tumors^1^. In our series, this sign was detected in all tumors and was not seen in the comparative group. So it can be considered as a distinctive CT feature of YST in clinical imaging practice. Intralesional vessels of YSTs usually have dilated lumen and marked enhancement. This classical imaging finding is considered to be a result of increased vascularity and the formation of small vascular aneurysms in the tumor^8^.

In our study, peritoneal spread, ascites or lymphadenopathy were detected in 80% of cases (8/10), which reflected the aggressive features of this type of tumor. Furthermore, the peritoneal metastatic lesions maintained their hypervascular characteristics with marked enhancement on images.

YST usually occurs in the second decade of life^9^. In our series, 7 patients were in their second decades and only 3 patients were over 20 years old (mean age 19.1 years). In women in their second decades, germ cell tumors are the most common histological type of ovarian tumors. Most of them are benign teratomas. But malignant germ cell tumors may be encountered. Among malignant germ cell tumors, immature teratoma is the most common and is followed by dysgerminoma and YST^10^. Imaging findings described for immature teratomas are predominantly cystic or solid cystic tumors and almost always contain fatty tissue^11^. Dysgerminoma include predominantly solid tumors associated with intratumoral fibrovascular septa, which show prominent enhancement. Necrosis and hemorrhage are rare occurrences^12^. These imaging findings are consistent to the cases in our study and are different from those obtained in YSTs.

Epithelial ovarian carcinomas represent 85% of malignant ovarian neoplasms. The two most common types are serous and mucinous cystadenocarcinomas^13^. Though their prevalence increases with age and peaks in the sixth and seventh decades of life, but some of them may still occur in young women, such as the patients reported in our study. On CT images, serous and mucinous cystadenocarcinomas were primarily cystic or solid cystic with solid tissue in varying enhancing degrees, and were associated with peritoneal implants, lymphadenopathy and ascites, mimicking an ovarian YST. It is quite difficult to differentiate these tumors from YST only according to these signs. However, enlarged intratumoral vessel, a specific characteristic of YST, was absent in this type of tumor. In our opinions, imaging findings with diagnostic value that are suggestive of cystadenocarcinomas include a thick, irregular wall; thick septa and papillary projections. Identification of papillary projections on imaging is important because they are single best predictors of an ovarian epithelial carcinoma^6^,^14^.

SLCT and SST, two types of ovarian sex cord-stromal tumors, are also commonly seen in young women (<30 years of age). SLCT is considered to be a low-grade malignancy which may be accompanied by ascites, abdominal or pelvic dissemination^15^. SST with ascites (Meigs’ syndrome) may appear like an ovarian malignancy on imaging^16^. So they also should be differentiated from YST. SLCT usually appears as a solid mass. A solid-cystic or cystic nature also can be seen on imaging. The mass in our study appeared as a well-circumscribed enhancing solid mass with intratumoral cysts on CT images, in agreement with the literature report^17^. These findings are considered to be the typical features of this type of tumor and are different from YST. In the present study, although the SST showed a predominantly solid mass with striking enhancement and ascites, the early and strong enhancement of the peripheral tumor tissue and its centripetal progression may help to diagnose this tumor, distinguishing it from YST. Furthermore, dilated intratumoral vessel are not found in SLCT and SST, necrosis and hemorrhage also have not been described in these two types of tumors in either the radiologic or the pathologic literature^15^,^16^,^17^,^18^.

In our series, Struma ovarii appeared as a multicystic mass with smooth surface and a high density component on precontrast scans, in agreement with the previous literatures^19^,^20^. Literatures report that Struma ovarii often occurs in the older population and the median age of diagnosis was 52 years^18^, although our patient was a young woman. The intratumoral high-density soft tissue on precontrast scans is the key for the differential diagnosis of Struma ovarii and YST. This high density component on precontrast scans may show marked enhancement or no enhancement on post-contrast scans. The solid component with marked enhancement, such as the case in our study, may represent thyroid tissue with dilated thyroid follicles. However, the solid component without obvious enhancement probably consists of viscid gelatinous colloid material^21^.

Bilateral YSTs are rare. Ayhan *et al*. have reported bilaterality rates of 8%^22^. In the current study, the bilaterality rate was 10%. For bilateral ovarian masses, Krukenberg tumors should be first considered and a careful search for the primary site should be made, especially the gastrointestinal tract. Another important disease to be distinguished from bilateral YSTs is serous cystadenocarcinoma which is 49.5% bilateral at time of diagnosis, and is following metastatic tumors (72%)^23^. However, lack of intratumoral vessels on imaging may help to distinguish bilateral serous cystadenocarcinomas from bilateral YSTs.

There are several limitations in our study. Firstly, this study was performed on a small series of cases. Secondly, different CT equipments and techniques were used. Thirdly, a detailed pathological study was absent to be correlated to the imaging findings. However, these problems are simply unavoidable due to the limitations of a retrospective study and the rarity of YST, and should not have significantly affected the imaging characteristics studied.

In summary, we present the largest cohort of radiological studies of ovarian YSTs to date. They are often seen as large, well-circumscribed, solid-cystic masses with intratumoral hemorrhage, marked heterogeneous enhancement and enlarged intratumoral vessels on CT images. For cases which are difficult to identify, carefully looking for dilated intratumoral vessels on post-contrast CT will be helpful to diagnose YST.

## Additional Information

**How to cite this article**: Li, Y.-K. *et al*. CT imaging of ovarian yolk sac tumor with emphasis on differential diagnosis. *Sci. Rep*. **5**, 11000; doi: 10.1038/srep11000 (2015).

## Figures and Tables

**Table 1:** Statistical analysis of the age and CT features between two groups.

	Patient (case)	Tumor (n)	Age (y)	Diameter (max, cm)	Shape (n)		Solid-cystic (n)	Hemorrhage (n)	Marked enhancement (n)	Intratumoral vessel (n)	Ascites (case)	Metastasis (case)
					Oval	Irregular						
YST	10	11	19.1 ± 4.3	13.9 ± 6.4	8	3	7	7	10	11	8	6
non-YST	16	18	21.2 ± 4.7	14.6 ± 9.1	7	11	5	4	9	0	13	7*
*P* value			0.252	0.807	0.082		0.023	0.033	0.029	0.000	0.657	0.473

^*^When calculating the difference of metastasis between 2 groups, 2 cases with benign tumors were excluded from non-YST group.

**Table 2:** Multivariate analysis of CT signs of yolk sac tumor.

	Odds ratio	95% CI	*P*-value
Solid cystic nature	6.627	0.975-45.059	0.053
Intratumoral hemorrhage	14.214	1.190-169.803	0.032
Marked enhancement	10.000	1.050-95.230	0.045
